# Modification of the Selectivity Properties of Tubular Ceramic Membranes after Alkaline Treatment

**DOI:** 10.3390/membranes7040065

**Published:** 2017-11-21

**Authors:** Patrick Dutournié, Lionel Limousy, Jérôme Anquetil, Sébastien Déon

**Affiliations:** 1 IS2M (UMR CNRS 7228), Université de Haute Alsace, 3 bis rue A. Werner, 68093 Mulhouse CEDEX, France; lionel.limousy@uha.fr; 2 TAMI-Industries, Z.A. Les Laurons CS65, 26111 Nyons CEDEX, France; janquetil@tami-industries.com; 3 Institut UTINAM Besançon (UMR CNRS 6213), Université de Bourgogne Franche-Comté, 16 Route de Gray, 25030 Besançon CEDEX, France; sebastien.deon@univ-fcomte.fr

**Keywords:** TiO_2_ membrane, pure salt-water filtration, polarisability, rejection sequence, dielectric effects

## Abstract

This work focuses on the selectivity modification of ceramic membranes after a mild alkaline treatment. Filtration of pure salt-water solutions was carried out with commercial titania membranes before and after the treatment. After treatment, the rejection of NaF significantly decreased, while the rejection of NaCl and NaBr increased. Additionally, NaI and Na_2_SO_4_ remained close to zero. Pore size and electrical charge being almost unchanged, only significant modifications in the dielectric effects can explain this modification of selectivity. Therefore, the surface chemistry and the interaction (nature and magnitude) with the solvent and with the species present in the solution appear to be modified by the alkaline treatment. This trend is also illustrated by discussing the electric and the dielectric properties that were numerically identified before and after treatment. The alkaline treatment significantly decreased the apparent dielectric constant of NaCl-water solution in the pore, highlighting the rejection of sodium chloride. Contrariwise, the modification of the surface chemistry increased the apparent dielectric constant of NaF-water solution by promoting fluoride transmission.

## 1. Introduction

Nanofiltration and low cut-off ultrafiltration are two separation processes widely used for industrial applications. These operations are very interesting for separation processes, because they require very little energy, they are easy to control, and they do not require additional chemicals, in contrast to other separation technics [1]. They are used in many fields (chemistry, the environment, medical, food, water production…) for different kinds of treatment (desalination, decontamination, rectification, separation, drug production…) [2,3,4,5]. These membranes include two major families of materials: organic materials (polymers) and ceramic materials (metal oxide, zeolite…). The first group is attractive thanks to the low cost and the possibility of being shaped into different configurations. On the other hand, the second family (ceramic membranes) is able to work in hard conditions (temperatures, pH, organic solvents, etc.) [6]. Many works have focused on the study of ceramic membrane performance [7,8,9,10]. The active layer of these membranes is often constituted by a metal oxide (Al_2_O_3_, ZrO_2_, TiO_2_), which presents amphoteric characteristics. This means that the surface charge of the active layer strongly depends on the pH of the solution. The presence of ionic species in the solution generates electrostatic interactions (attraction or repulsion), which may be at the origin of ion adsorption and, subsequently, of the modification of membrane properties [11]. These membranes can be prepared by the sol-gel method, but also by molecular layer deposition (in the case of TiO_2_). They allow to very thin filtration layers, high flux and high selectivity (average pore size ≈ 1 nm) to be obtained [12]. TiO_2_ is used in a wide range of applications, ranging from paint formulation to solar cells; it can be easily synthesized, and can be present in four different fundamental crystal forms (anatase, rutile, brookite and TiO_2_) [13]. Several works have recently published reports on the use of this material for the production of commercial titanium dioxide membranes, and on the membrane performances [14,15,16]. These studies mainly focused on the anti-fouling properties of TiO_2_ due to its hydrophilicity [14], its adsorption equilibrium, and the electrostatic interactions of TiO_2_ with different ionic solutes (Ca^2+^, Mg^2+^, PO_4_^3−^, etc.) [15,16]. The works available in the literature deal with the filtration of ionic solutions (most commonly mixtures) for particular operating conditions (pH, temperature…). Explanations of the selectivity of these membranes are often limited to a discussion of the steric and electric effects. Often, the dielectric effects are neglected, because authors did not take into account the confinement of the solution inside the pore due to the small pore diameter of the TiO_2_ membranes (low cut-off ultrafiltration membranes with pore diameters of between 2 and 4 nm). Moreover, only a few studies have focused on the rejection of pure salt-water solutions [15], and none have investigated the filtration properties before and after a mild alkaline treatment of the filtration layer surface. More generally, few works are devoted to the property modifications after a treatment. Indeed, these studies have focused on the hydraulic performances and antifouling properties. For example, Albo et al. [17,18,19] studied the water and IPA performances with polyamide membranes for pervaporation and vapor permeation after three different pre-treatments. They observe an increase of the water permeance for membranes dried with ethanol-hexane. Other studies (see Ref. [20]) reported on the addition of nanoparticles (or nanotubes) to polymers during membrane synthesis, or on the synthesis of ceramic membranes with catalytic nanoparticles, for preventing bio-fouling by modifying the surface hydrophilicity. For example, Yang et al. [21] observed an increase of hydraulic performances of a TiO_2_-doped alumina membrane compared to the alumina homogeneous membrane, owing to a modification of hydrophilic properties. Dutournié et al. [22] observed a decrease of the hydraulic performances of a zeolitic membrane after a chemical treatment. Contact angle investigations have shown that the decrease of membrane hydraulic performances is correlated with an increase of surface hydrophilicity. In addition, Y. Zhu et al. [23] examined the recent development of polymeric and ceramic membranes (synthesis and surface treatment) in terms of liquid flux and antifouling properties for emulsified oil/water separation. Nevertheless, few or no studies have investigated the effect of surface treatment in terms of ionic selectivity. However, the understanding of the relationship between surface chemistry and membrane selectivity for ionic solutes is strategically important for modeling membrane performances, for scaling-up, and for improving manufacturing process intensification. Indeed, current knowledge of phenomena that govern mass transfer in microporous materials is very limited and do not allow to explain the behavior observed in various case studies [24,25]. The main aim of this work is to understand and interpret the selectivity performances of commercial titania membranes. These membranes were selected because their pore size is large enough not to be modified by an alkaline treatment. Then, it is possible to neglect the modification due to steric effects after a mild alkaline treatment. Moreover, the hydraulic performances of such membrane materials are stable over time (no evolution of the hydraulic permeability), and remain constant after mild alkaline treatments.

## 2. Materials and Methods

First, three titanium dioxide membranes provided by TAMI Company (Nyons, France) were used in this study (internal diameter = 8 mm, length = 25 cm, classification = 1 kDa). They consisted of a macroporous tubular support (*α*-alumina) presenting three different porosities (varying from 5 µm for the external part to 0.2 µm for the internal surface), and coated with a thin layer of TiO_2_ (of 2 µm approximately). Titanium dioxide powder coming from the same batch as that for the membranes used was provided by TAMI Company (Nyons, France) for additional investigations.

Observation of the membrane surface was performed by scanning electron microscopy (Philips XL30 FEG, SEMTech Solutions, North Billerica, MA, USA) to characterize the thickness and the roughness of the TiO_2_ filtration layer. For this purpose, a membrane was broken to observe the interface between the TiO_2_ active layer and the support (macroporous alumina).

Zetametry experiments were conducted with a Zetasizer instrument (Nano ZS, Malvern Instrument, Orsay, France). 50 mg of TiO_2_ powder was introduced into 50 mL of the studied salt solutions (5 × 10^−3^ M). The pH was adjusted by addition of HCl or NaOH solutions (0.1 M). The different suspensions were dispersed by ultrasound treatment in order to avoid the presence of agglomerates. The suspensions were then shaken overnight to guarantee the ionic equilibrium.

Hydraulic and selectivity performances of the different membranes were investigated with a laboratory pilot-plant previously described [19]. The tubular membrane was placed in a stainless steel carter. The feed solution was poured into a 5 L tank, and a volumetric pump provided the feeding of the solution. The flow rate was controlled by an analog sensor, and kept at 700 L/h. The transmembrane pressure was adjusted between 4 and 12 bar by a manual valve, and was continuously measured by two analog sensors placed upstream and downstream of the membrane. The temperature of the fluid was maintained at 25 °C by a cooling unit. Filtration tests were performed in closed-loop by flowing back the permeate into the feed solution after sampling and analysis. Pure water filtration tests were performed with demineralized water (conductivity lower than 0.1 µS/cm). Filtration tests of pure salt-water solution were performed with NaF, NaCl, NaBr, NaI and Na_2_SO_4_ salts (Sigma-Aldrich, St. Quentin Fallavier, France, purity > 99% for each). Permeate and retentate samples were weighed and analyzed with a conductimeter (GLP 31, Crison, Estella, Spain) to estimate the permeation flux and salt concentrations on both sides of the membrane. Filtration tests of a neutral organic solute (Vitamin B12) also carried out to estimate the main pore diameter of the membrane. Retentate and permeate concentrations were estimated by absorbance measurements at 362 nm with spectrophotometer Lambda 35 (Perkin Elmer Instrument, Waltham, MA, USA).

Stabilization of the membrane properties was carried out before performing the ionic filtration tests. This conditioning step is requisite and useful for reaching steady membrane performances [8]. It consists of the filtration of pure water at constant operating conditions (flow rate, temperature and applied pressure of 5 bar). During this step, the hydraulic performances of the membrane decrease rapidly, due to surface hydration and particle reorganization, and are characterized by a slow and progressive decrease in performance due to microporous hydration [26]. After membrane conditioning, the hydraulic performances were monitored to ensure membrane stability. Between each experiment, a filtration test of pure water was performed. This consisted of the measurement of the water flux (*J_w_*) for different applied pressures. The hydraulic permeability (*L_p_*) was then obtained via Equation (1):

(1) $$\mu Jw=L_{p}\Delta P$$

A chemical treatment was carried out to modify the membrane selectivity properties. It was performed by filtration of a sodium carbonate-water solution (6.6 mM, pH = 10.5) over two hours. After the alkaline treatment, the membrane was thoroughly rinsed with demineralized water.

All ionic solutions (pure salt-water solution) contained a common ion (Na^+^) at the same concentration (5 mM). The solution was introduced in the filtration unit 12 h before filtration tests to reach the ionic equilibrium in the pores. To limit or avoid polarization concentration phenomena, the salt concentration was maintained at a low value (5 mM), and the operating flow rate was set 700 L/h providing a turbulent flow (Re = 23,000, U∞ ≈ 5 m·s^−1^), consequently the salt concentration at the membrane wall was the same as that of the feed solution.

The observed rejection rates *R_i_*, were estimated by using Equation (2) and plotted as a function of the permeation flux.

(2) $$R_{i}=\frac{C_{i,r}-C_{i,p}}{C_{i,r}}$$

Experimental errors:

- Zetametry measurements: all tests were repeated three times, the pH was measured with a numerical pHmeter (+/− 0.01), the observed maximal Zeta potential error was 3.6 mV.
- Rejection experiments: the permeation flux was calculated from the mass (error ≤ 0.2%) and the time measurements (error ≤ 1.7%), with a relative error less than 2%. Neutral solute rejection (Vitamin B12) was calculated from absorbance measurements according to Equation (3). The results are given in Table 1.

Additional tests performed in parallel with different apparatus showed that the relative error is less than 3%.

(3) $$\Delta R=\frac{Cp}{Cr²}\Delta Cr+\frac{1}{Cr}\Delta Cp$$

The experimental error for ionic solutes rejection rate was calculated from conductivity measurements (errors < 10 µS/cm) by Equation (3). The results showed that the rejection maximal error was lower than 3% in all studied cases. Nevertheless, these calculations do not take into account eventual holistic errors, which are certainly higher (wait for steady state, perfect rinsing of membrane carter...).

## 3. Modeling Part

The model used in this study for interpreting the experimental results is accurately detailed in previous works [27,28,29]. It simulates the transmission of ions through the membrane by modeling the ion transport within the pores (using the extended Nernst-Planck approach). The flux of each ion is described by the sum of diffusion, convection and electro-migration contributions (Equation (4)), as follows:

(4) $$j_{i}\left(x\right)=-c_{i}K_{i,d}D_{i,\infty }\frac{d\left[\ln\gamma_{i,p}\right]}{dx}-K_{i,d}D_{i,\infty }\frac{dc_{i}}{dx}-\frac{z_{i}c_{i}K_{i,d}D_{i,\infty }}{RT}F\frac{d\psi }{dx}+K_{i,c}c_{i}V$$

This differential equation is completed by two boundary conditions: equilibrium partitioning, which is the equality of generalized chemical potentials on both sides of the membrane-solution interfaces. The scientific community agrees three three phenomena act on the ion separation: the steric, the electric, and the dielectric effects. From these assumptions, the equilibrium partitioning can be written (Equation (5)):

(5) $$\frac{c_{i}}{C_{i}}=\frac{\gamma_{i,s}}{\gamma_{i,p}}\phi_{i}\exp\left(-\Delta W_{i}\right)\exp\left(\frac{-z_{i}F}{RT}\Delta \psi_{{}_{D}}\right)$$

The ratio between ion activities on both sides of the interface is the product of a steric (*φ_i_* = ${\left(1-\frac{r_{{}_{S}}(i)}{r_{p}}\right)}^{2}$), an electric (described by the Donnan potential: $\Delta \psi_{{}_{D}}$), and a dielectric contributions ($e^{-\Delta W_{i}}$). This last contribution is estimated by approximating the dielectric exclusion of a decrease of an apparent solvent permeability using Equation (6):

(6) $$\Delta W_{i}=\frac{z_{i}^{2}e^{2}}{8\pi \varepsilon_{0}k_{B}Tr_{{}_{S}}(i)}\left(\frac{1}{\varepsilon_{p}}-\frac{1}{\varepsilon_{b}}\right)$$

The volumetric permeation flux can be calculated from Equation (7):

(7) $$J_{v}=\frac{L_{p}}{\mu }\left(\Delta P-\Delta \pi \right)$$

To simulate membrane performance, four intrinsic parameters are needed: membrane permeability *L_p_*, which is assessed by pure water filtration tests (Equation (1)); mean pore radius *(r_p_*); electric charge in the pore *X_d_* and the apparent dielectric constant in the pore *ε_p_*.

Size exclusion was investigated by studying the rejection of a neutral solute (filtration of Vitamin B12-water solution, 100 mg/L). The average pore radius was calculated by fitting the rejection data of vitamin B12 by an algebraic equation derived by the Nernst-Planck approach for uncharged solutes. This equation is derived from the solution of the 1D differential equation of mass transport in the porous media by assuming that the pores are uniformly dispersed, possess only one size, and are cylindrical. The boundary condition is the equality of chemical potentials at the pore/solution interface, considering only steric effects [20]. After estimation of membrane permeability and average pore radius, the numerical program was used to estimate all the values (*X_d_*, *ε_p_*), providing the best fits of the experimental rejection curves.

## 4. Results and Discussion

### 4.1. Surface Properties

**Figure 1:**
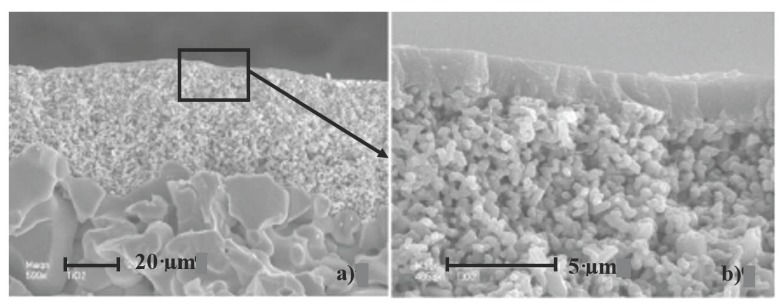
SEM images of the cross-section of a TiO_2_ membrane at different enlargements. (**a**) magnification of 500×, macroporous alumina at the bottom, mesoporous alumina in the middle and TiO_2_ layer at the top; (**b**) Zoom of the selected area at a magnification of 4600×, thin layer of TiO_2_ at the top and mesoporous alumina just below.

SEM investigations were performed on various pieces of a membrane. Figure 1 shows a cross-section observation of the tubular membrane at two different enlargements. First, the micrographs show that the membrane support (*α*-alumina) is constituted of several layers with different porosities. The active layer in titanium dioxide thickness was between 1 and 2 µm (top layer on Figure 1b).

**Figure 2:**
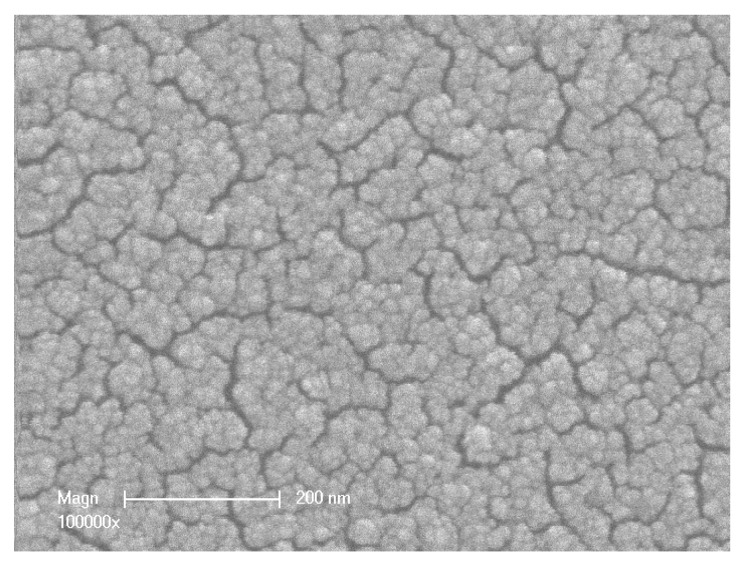
SEM photograph of the membrane surface (top view of the active layer ×100,000).

The following micrograph (Figure 2) shows a top view of the membrane active layer. This figure shows that the active layer is constituted by an agglomerate of TiO_2_ nanoparticles. This agglomeration is certainly associated with particle sintering arising during thermal treatment.

The electric surface charge of the membrane was investigated by zetametry measurements [30]. The measurements were performed with TiO_2_ powder provided by TAMI Company, basically identical to the TiO_2_ constituting the active layer of the studied membranes (TiO_2_ powder from the same batch as the membranes used in the present study).

**Figure 3:**
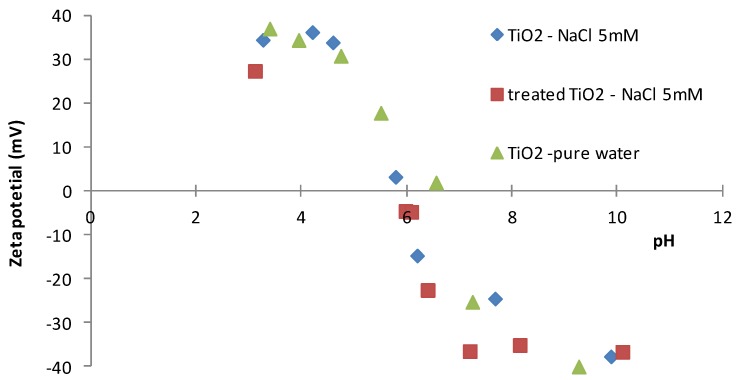
Zetametry results of TiO_2_ powder (before and after Na_2_CO_3_ treatment) in a NaCl (5 mM)—water solution.

Zetametry investigations showed (Figure 3) that the electrical charge of the membrane did not change after the alkaline treatment for the TiO_2_ powder in NaCl-water solution. The isoelectric point of TiO_2_ is around pH = 6, whatever the treatment performed, and only a slight decrease of pH was observed when NaCl was in contact with the surface of TiO_2_. These results are approximately the same than those obtained with TiO_2_ powder in pure water. These results indicate that, during filtration tests (carried out at a pH close to 7), the surface of the membrane was negatively charged, and that NaCl did not affect the surface charge of the membrane.

**Figure 4:**
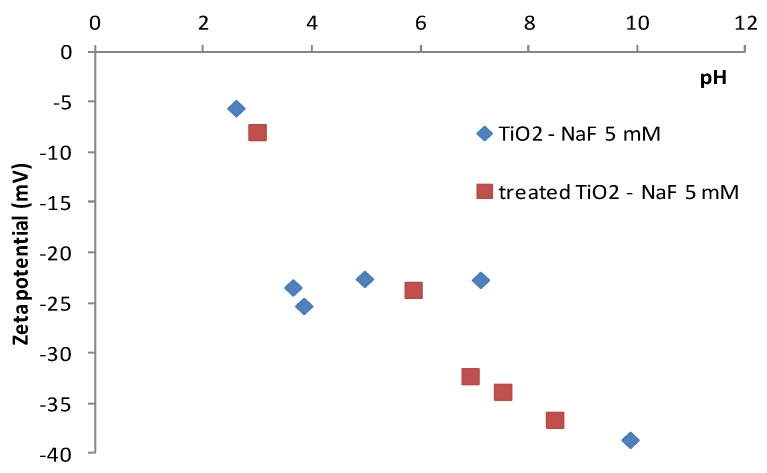
Zetametry results of TiO_2_ powder (before and after Na_2_CO_3_ treatment) in a NaF (5 mM)—water solution.

Figure 4 shows the zetametry results obtained after contact between TiO_2_ particles and the NaF–water solution. We can observe that the isoelectric point of TiO_2_ was strongly affected in the presence of NaF; from pH = 6.5 for pure water to pH ≈ 2 for the NaF water solution. This modification may be attributed to a strong interaction between the fluoride ion and TiO_2_, which induces a negative charge at the surface of the particles even for a pH below 6.5. As for the previous results, the electrical properties of the TiO_2_ powder were identical for the treated and non-treated samples. This result confirms that the alkaline treatment did not modify the surface charge of TiO_2_. When NaF was present in the solution, the membrane charge remained fully negative for a large range of pH. To conclude, the alkaline treatment did not modify the electrical charge of the filtering material, but the electric charge of its surface may have been strongly affected by the presence of NaF.

### 4.2. Hydraulic Performances, Average Pore Radius

After membrane conditioning, a filtration test of pure water was performed to evaluate the membrane hydraulic performances. After this test, the rejection of a neutral solute was studied to estimate the apparent pore size. Table 1 summarizes the hydraulic permeabilities after stabilization, and the filtration performances of a neutral solute (Vitamin B12) for the three studied membranes.

The hydraulic permeabilities and the rejection of VB 12 are similar for membranes 1 and 2. Membrane 3 shows better hydraulic performances with a higher mean pore radius than membranes 1 and 2. The estimation of mean pore radius via the Nernst-Planck approach for uncharged solutes [27,31] gives values in the 1.3 to 1.5 nm range for membranes 1 and 2, respectively, and of 2.3 nm for membrane 3. It is important to note that this methodology leads to an overestimation of the average pore radius. Indeed, taking pore size distribution into account leads to a decrease of the estimated average pore radius. An interesting observation is that pore radii of the different tested membranes are very large compared to the radii of the studied ions.

### 4.3. Pure Salt-Water Filtration Experiments

Filtration tests of pure salt-water solutions were carried out on the three membranes. Before each experimental test, a filtration test of pure water was performed to verify the stability of the hydraulic performances of the different membranes.

**Figure 5:**
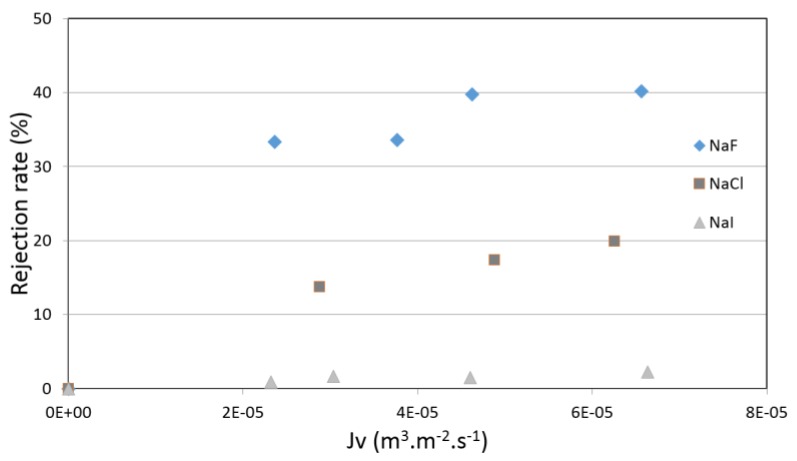
Rejection rate of pure salt-water solution (NaF, NaCl or NaI—5 × 10^−3^ M) vs. permeation flux obtained with membrane 2.

**Figure 6:**
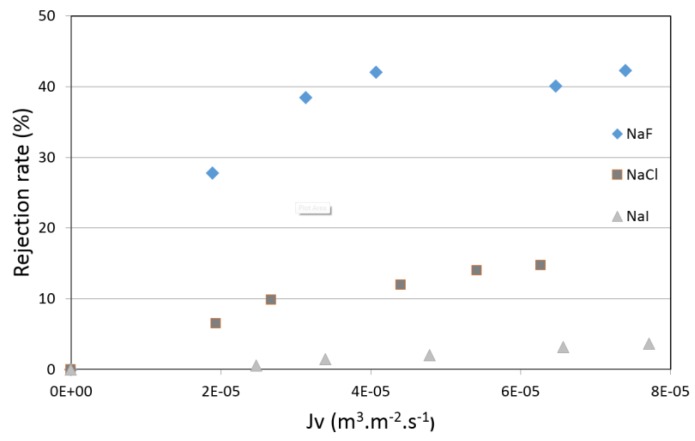
Rejection rate of pure salt-water solutions (NaF, NaCl or NaI—5 × 10^−3^ M) vs. permeation flux obtained with membrane 3.

Figure 5 and Figure 6 show the rejection rates of pure salt-water solutions as a function of the permeation flux for membranes 2 and 3. The first finding is that the rejection rates of pure halide salts were almost the same for membranes 2 and 3. This observation is important, because two membranes with very different pore radii can separate in a similar way to halide salts (around 40% for NaF, 20% for NaCl and <5% for NaI), which was not the case for neutral molecules (60% vs. 33%). Another observation is the difference of rejection rates between the three studied salts. NaF is more retained than NaCl, whereas NaI is fully transmitted. The Stokes radii of halide ions (0.166, 0.121 and 0.120 nm for F-, Cl- and I-respectively) [32] cannot explain the observed sequence of rejection, and suggest that, in the present case, steric effects are not the determining phenomenon for exclusion. Indeed, the scientific community agrees that the selectivity of ionic species is governed by three phenomena: steric, electric and dielectric. In this case, it can be assumed that the steric effects cannot be responsible of these experimental rejections. This means that a combination of electric and dielectric effects might be at the origin of the partial rejection of these ions.

The filtration results of pure salt-water solution with the 3 membranes are summarized in Table 2. As shown by Song et al. [12] in a recent work, if only the Donnan exclusion is at the origin of the salt rejection, sulfate ions must be more rejected than chlorides by such a type of membrane. As observed in Table 2, the opposite results were obtained with the three TiO_2_ membranes. These results clearly indicate that other phenomena are at the origin of the membrane performance in regard to ion rejection.

The results presented in Table 2 confirm the previous observations. An additional test performed with membrane 2 (filtration of a sodium bromide-water solution, 5 mM) shows that the rejection order is R (NaF) > R (NaCl) ≈ R (NaBr) > R (NaI) for halide salts. This result requires additional explanation to the classic ones (steric effect, electric effect…). Another interesting result is the transmission of sodium sulfate, which is almost complete. The divalent anion presents two negative charges and, looking to the membrane electric surface charge (negative whatever is the solution), the repulsion phenomena should be more important, but such a trend is not observed. Except for the sulfate ion, the rejection rates of the studied ions are in agreement with the Hofmeister series (R (NaF) ≈ R (Na_2_SO_4_) > R (NaCl) > R (NaBr) > R (NaI)) [33]. In the same way, the hydration energies (ΔH_hyd_ (Na_2_SO_4_) > ΔH_hyd_ (NaF) > ΔH_hyd_ (NaCl) > ΔH_hyd_ (NaBr) > ΔH_hyd_ (NaI)) of the different ions are in good agreement with the transmission sequence, except for sulfate ions. The investigation of the electric properties of TiO_2_ showed that, in the studied range of pH, the active filtration layer was negatively charged. Because all the salts studied present a common concentration of Na^+^ ion, even if the adsorption process occurs in the pore, the electrical charge (due to the material surface charge and the adsorbed species) cannot change significantly, except for fluoride.

From this point of view, it seems that dielectric effects are the main phenomena acting on separation. These effects are described in various ways in the literature [34,35,36]. They are the consequence of confinement in the pore, the difference of dielectric constant between the fluid and the material, the deformation of electrical yield lines in the immediate vicinity of surface charges or polar groups, the nature of the solvent, and the nature of ions in solution [37]. In the present work, all the parameters are the same (nature of the compensation cation, ion concentrations, active layer, confinement…) except the nature of the negative ions and their interactions with the surface of TiO_2_. To study these interactions, rejection sequences of ions are investigated with regard to the ion polarizabilities. This property is the ability of an ion to relocate its electrical charge(s) to minimize interaction energy with its environment. The polarizabilities of the studied ions are: F^−^(0.8 Å3), Cl^−^(3 Å3), Br^−^(4.2 Å3), I^−^(6.5 Å3) and SO_4_^2−^(7 Å3) [38,39]. The rejection sequence is in agreement with the polarizabilities of the negative ions. A previous study performed with a zeolitic membrane [40] had shown a correlation between membrane selectivity and ion polarizabilities.

### 4.4. Filtration Experiments after Alkaline Treatment

After filtration of a sodium carbonate-water solution during 2 h (pH = 10.5) and abundant rinsing, the filtration tests were repeated in the same order.

The hydraulic and steric properties of the membrane, before and after the alkaline treatment, are given in Table 3. It seems that the hydraulic membrane permeabilities slightly increased after the treatment. The filtration tests of Vitamin B12, surprisingly, showed an increase of the permeability, which could be easily interpreted as an increase in the pore radius (at constant pore number). In the present case, the average pore radius tended to slightly decrease after the alkaline treatment.

Table 4 compares the filtration performances of the three studied membranes before and after the treatment. First, the sodium sulfate was mainly transmitted by the three membranes either before or after the alkaline treatment. It seems that the treatment had no influence on the sulfate ion rejection. In contrast, rejection rates of fluoride, chloride and bromide ions were clearly modified. The transfer of chloride and bromide ions was restrained, whereas fluoride ion transmission was increased.

**Figure 7:**
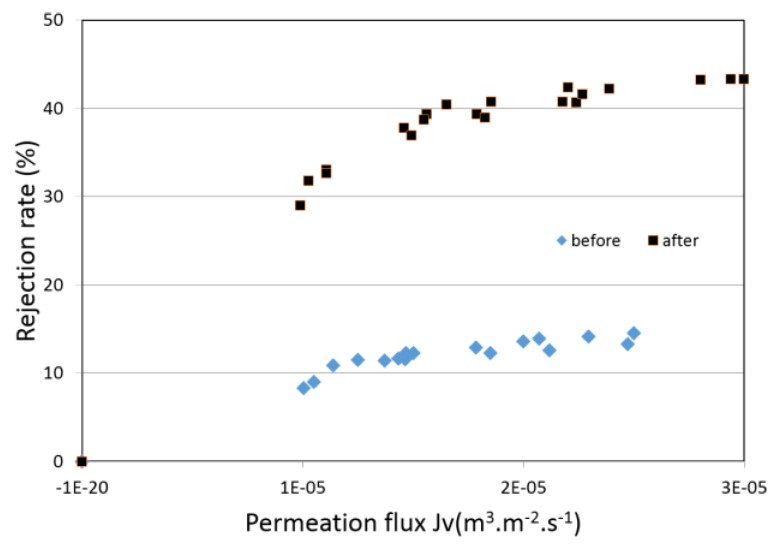
Rejection rates of NaCl-water solutions vs. permeation flux (membrane 1).

Figure 7 shows the rejection rates observed for filtration of sodium chloride-water solutions before and after the chemical treatment. To ensure the stability of the membrane performances, filtration tests of sodium chloride-water solution were repeated between the tests performed with the other salts. The two experimental series are the data compilations of 8 filtration tests (the time required for the test series was more than one month). These tests show firstly that the membrane performances were stable, and the measurements reliable. Moreover, the rejection rate of sodium chloride was three times larger after the alkaline treatment.

**Figure 8:**
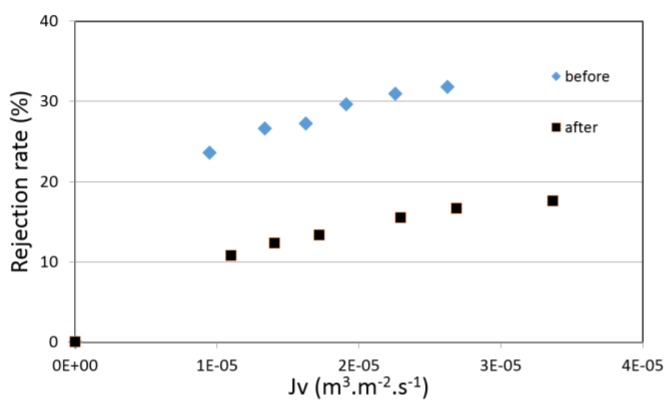
Rejection rates of NaF-water solutions vs. permeation flux (membrane 1) before and after the alkaline treatment.

Figure 8 shows the observed rejections of sodium fluoride before and after the treatment. In contrast to the previous results obtained with sodium chloride solutions, the rejection rates of fluoride ions were halved after alkaline treatment.

The different investigations performed showed that the membrane hydraulic performances were slightly modified after the alkaline treatment. Following the same trend, the steric contribution on the ionic transport phenomena was weak or completely absent. Significant modifications of the electric and dielectric effects can only explain the observed results. The results concerning the transmission of sulfate ions and the zetametry investigation, performed before the treatment, led us to think that the electric effects do not strongly contribute to retention performance; it seems that the dielectric effects are finally the major contribution to the ionic mass transfer in such types of membrane. To resume, before the treatment, the obtained results were able to be explained by electric and dielectric effects. As the electric properties of the material did not change after treatment, only a significant modification of dielectric effect was able to explain the observed results. The increase of hydraulic properties of the membranes after the alkaline treatment can be explained by a decrease of the surface hydrophilicity [22]. The fluoride ions in the water were less deformable, and the interaction energy with a hydrophilic surface was maximal. After the treatment, this energy decreases, ensuring an increased transmission of the salt.

**Figure 9:**
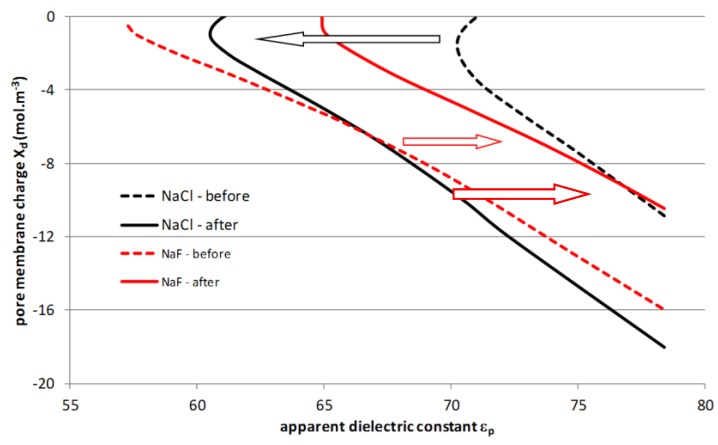
Best-fitted values (*X_d_*, *ε_p_*) for the numerical approximation of rejection curves.

This explanation is physically investigated by assessing the various couples (*X_d_*, *ε_p_*) that fit the experimental rejection curves before and after treatment. It should be underlined that an infinite number of couples can be identified to describe the rejection curves and the values reported in Figure 9.

Although the right couple cannot be determined from pure salt solutions, Figure 9 enables a relevant quantitative discussion. Indeed, if the charge density inside the pores is assumed to be very weakly impacted by the alkaline treatment, the simulations show that the dielectric constant is necessarily modified by the treatment. In the case of NaCl, the identified dielectric constant tends to be decreased by the treatment (higher dielectric exclusion), while the value increases for NaF, leading to lower dielectric exclusion. It is obvious that the selectivity order inversion between these two salts is probably due to both phenomena, but dielectric contribution is probably the predominant mechanism.

## 5. Conclusions

The filtration properties and the performances of three commercial membranes were studied. Filtration tests of different pure salt-water solutions with a common ion were performed before and after an alkaline treatment. Before the treatment, the filtration tests of the ionic solutions show the following rejection order NaF > NaCl ≈ NaBr > Na_2_SO_4_ > NaI. From these results, and taking into account the various characterizations performed, we can state that steric and electric effects alone cannot explain such an order of rejection. Only the dielectric effects are able to partially explain these results, especially according to the ion polarizabilities. After the alkaline treatment, the rejection rates of sodium fluoride and sodium chloride are completely modified. The rejection rate of fluoride ions is decreased by half, while that of chloride ions is significantly increased. At the same time, sulfate and iodide ions are fully transmitted (before and after the treatment). A modification of the interactions between the TiO_2_ surface and the different ions after the alkaline treatment is at the origin of these modifications. Even though the numerical simulations confirmed the modification of the dielectric exclusion by the treatment, further investigations need to be done in order to understand the nature of these modifications and the behavior of chloride and bromide ions when compared to fluoride ions.

## Figures and Tables

**Table 1:** Hydraulic permeabilities, rejection rate of Vitamin B12, and estimated mean pore radius of the different TiO_2_ membranes.

Membrane Performances	Membrane 1	Membrane 2	Membrane 3
10^14^ *Lp* (m^3^·m^−2^_memb_)	2.95	3.8	5.7
Rejection rate (%)	69 (±1.9)	60 (±2.4)	33 (±4.0)
Average pore radius (nm)	1.4	1.5	2.3

**Table 2:** Maximal rejection rates obtained with membranes 1, 2 and 3 for all the different filtration tests carried out with pure salts ([Na^+^] = 5 × 10^−3^ M for all the solutions).

Maximal Rejection Rate (%)	Membrane 1	Membrane 2	Membrane 3
NaCl (5 mM)	14	20	15
NaF (5 mM)	32	40	42
NaI (5 mM)	≈0	2	3
Na_2_SO_4_ (2.5 mM)	10	7	4
NaBr (5 mM)	--	17	--

**Table 3:** Hydraulic permeabilities, rejection rate of Vitamin B12, and estimated mean pore radius before and after the alkaline treatment.

Membrane Performances	Membrane 1		Membrane 2		Membrane 3	
	before	after	before	after	before	after
10^14^ Lp (m^3^·m^−2^_memb_)	2.95	3.1	3.8	4.3	5.7	6.0
VB 12 rejection rate (%)	69	--	60	56	33	56
Average pore radius (nm)	1.4	--	1.5	1.6	2.3	1.6

**Table 4:** Maximal rejection rates obtained with membranes 1, 2 and 3 for all the studied solutions before and after the alkaline treatment.

Maximal Rejection Rate (%)	Membrane 1		Membrane 2		Membrane 3	
	before	after	before	after	before	after
NaCl (5 mM)	14	42	20	35	15	36
NaF (5 mM)	32	18	40	32	42	26
NaI (5 mM)	0	0	2	3	3	8
Na_2_SO_4_ (2.5 mM)	10	8	7	7	4	2
NaBr (5 mM)	--	--	17	29	--	--

## Glossary

*c**_i_*	concentration of ion i within the pore (mol·m^−3^)
*C**_i,p_*	permeate concentration of ion i (mol·m^−3^)
*C**_i,r_*	bulk concentration of ion i (mol·m^−3^)
*D**_i,∞_*	molecular diffusion coefficient of ion i at infinite dilution (m^2^·s^−1^)
*e*	electronic charge (1.602 × 10^−19^ C)
*F*	Faraday constant (96,487 C·mol^−1^)
*j**_i_*	ionic flux of ion i (mol·m^−2^·s^−1^)
*J**_v_*	volumetric permeation flux (m^3^ m^−2^·s^−1^)
*J**_w_*	volumetric permeation flux of pure water (m^3^·m^−2^·s^−1^)
*k**_B_*	Boltzmann constant (96,487 C·mol^−1^)
*K**_i,c_*	ionic hindrance factor for convection (dimensionless)
*K**_i,d_*	ionic hindrance factor for diffusion (dimensionless)
*L**_p_*	water permeability (m^3^·m^−2^)
*P*	pressure (Pa)
*R*	universal gas constant (8.314 J·mol^−1^·K^−1^)
*r**_S_*(*i*)	Stokes radius of ion i (m)
*R**_i_*	observed rejection of ion i (dimensionless)
*r**_p_*	average pore radius (m)
*T*	temperature (K)
*V*	solvent velocity in the pore (m·s^−1^)
*x*	axial position within the pore (m)
*X**_d_*	membrane effective charge density (eq·m^−3^)
*z**_i_*	valence of ion i (dimensionless)
Greek Letters	
*γ_i,p_*	activity coefficient of ion i in the pore (dimensionless)
*γ_i,s_*	activity coefficient of ion i in the solution side of the interface (dimensionless)
Δ*P*	applied pressure (Pa)
Δ*W_i_*	dielectric exclusion energy (J)
Δ*y_D_*	Donnan potential (V)
Δ*p*	osmotic pressure difference (Pa)
*ε_0_*	permittivity of free space (8.85419 × 10^−12^ F·m^−1^)
*ε_b_*	bulk dielectric constant (dimensionless)
*ε_p_*	pore dielectric constant (dimensionless)
*µ*	dynamic viscosity (Pa·s)
*φ_i_*	steric partition coefficient (dimensionless)
*ψ*	electrical potential within the pore (V)
Subscript	
*i*	ion i
*p*	pore
